# Following the science? Views from scientists on government advisory boards during the COVID-19 pandemic: a qualitative interview study in five European countries

**DOI:** 10.1136/bmjgh-2021-006928

**Published:** 2021-09-27

**Authors:** Elien Colman, Marta Wanat, Herman Goossens, Sarah Tonkin-Crine, Sibyl Anthierens

**Affiliations:** 1 Department of Family Medicine and Population Health, University of Antwerp, Antwerpen, Belgium; 2 Nuffield Department of Primary Care Health Sciences, University of Oxford, Oxford, UK; 3 Laboratory of Medical Microbiology, Vaccine and Infectious Disease Institute, University of Antwerp, Antwerp, Belgium; 4 NIHR Health Protection Research Unit in Healthcare Associated Infections and Antimicrobial Resistance, University of Oxford in Partnership with Public Health England, Oxford, UK

**Keywords:** COVID-19, health policy

## Abstract

**Introduction:**

In order to tackle the pandemic, governments have established various types of advisory boards to provide evidence and recommendations to policy makers. Scientists working on these boards have faced many challenges, including working under significant time constraints to produce ‘evidence’ as quickly as possible. However, their voices are still largely missing in the discussion. This study explores the views and experiences of scientists working on government advisory boards during the COVID-19 pandemic, with the aim to learn lessons for future pandemic management and preparedness.

**Methods:**

We conducted online video or telephone semi-structured interviews between December 2020 and April 2021 with 21 scientists with an official government advisory role during the COVID-19 pandemic in Belgium, the Netherlands, UK, Sweden and Germany. The interviews were audio-recorded and transcribed and analysed using a combination of inductive and deductive thematic analysis techniques.

**Results:**

Scientists viewed the initial focus on biomedically oriented work during the pandemic as somewhat one-dimensional, but also highlighted difficulties of working in an interdisciplinary way. They found it difficult at times to ensure that the evidence is understood and taken on board by governments. They found themselves taking on new roles, the boundaries of which were not clearly defined. Consequently, they were often perceived and treated as a public figure.

**Conclusion:**

Scientists working on advisory boards in European countries faced similar challenges, highlighting key lessons to be learnt. Future pandemic preparedness efforts should focus on building interdisciplinary collaboration through development of scientists’ skills and appropriate infrastructure; ensuring transparency in how boards operate; defining and protecting the boundaries of the scientific advisor role; and supporting scientists to inform the public in the fight against disinformation, while dealing with potential hostile reactions.

**Key questions**

**What is already known?**

- Scientists have played key role in providing scientific advice to governments during the COVID-19 pandemic.
- With science becoming a focal point of this pandemic, scientific advisors also found themselves in the public eye.
- The views of key actors, that is, government scientific advisors, are still largely missing and they are crucial for understanding what we can learn from this pandemic and how we can prepare for the next one.

**What are the new findings?**

- Scientific advisors working during the COVID-19 pandemic faced a number of challenges, such as working in an interdisciplinary way with their peers on scientific boards, establishing a working relationship with the government and facilitating the process of evidence to be taken on board, and dealing with media and public reactions.
- Scientists found themselves taking on new roles, the boundaries of which were not clearly defined.

**What do the new findings imply?**

- Looking for entry points where other disciplines such as behavioural, social and political sciences, engineering, and economics can bring added value to some of the more clinical or biomedically oriented work can improve advisory boards’ preparedness to perform their expert roles during future crises.
- There is a need for a better clarity around the role of scientific advisors and the distinction between scientific advice and government decisions for all actors, including policy makers, media, the public and the scientific advisors themselves.
- Scientific uncertainties need to be explicitly assessed and communicated transparently to the public in order to maintain trustworthiness and facilitate public trust in science.

## Introduction

Since January 2020, when the WHO has declared COVID-19 to be a global healthcare emergency,^1^ countries across the world have witnessed its devastating consequences. In order to tackle the pandemic, governments have established various types of scientific advisory boards to provide evidence and recommendations to policy makers.^2–4^ These scientists have been faced with numerous challenges, including working under significant time constraints to produce evidence as quickly as possible,^5 6^ and taking on new roles, such as interacting with the media and the public or giving policy recommendations in unprecedented ways, and being in the spotlight. While scientists have been able to make some impact on policy since the start of the pandemic in European countries,^7–9^ the complex relationship between scientists and governments during this pandemic has been widely discussed. There have been debates about the boundaries, scope and neutrality of the role of scientific advisors, which highlighted the complexity of the advisory process, with some questioning whether it is even possible for scientists to provide value-free recommendations.^3 9 10^ There have also been reports of policy makers’ attempts to influence the scientific advisory boards, for example, in the UK,^10^ Belgium^11^ and the Netherlands,^12^ which highlighted at times blurred boundaries of the government–scientist relationship.

During the COVID-19 pandemic, the statement of ‘following the science’ has been used by politicians as both a shorthand for a new, ‘better’ era of politics, where government decisions will be based on scientists’ and public health experts’ advice, for example in the USA,^13^ as well as an explanation or even excuse for certain government’s decisions and failings related to handling this pandemic.^8^ However, some highlighted that the assumption behind this statement and traditional positivistic view and understanding and use of evidence-based medicine (EBM), namely that we can access singular truth through empirical enquiry and the use of clearly defined hierarchy of methods, should in fact be contested.^14^ While these debates are not new (eg, refs 15 16), some argued that the COVID-19 pandemic has brought the urgency of challenging our understanding and use of EBM.^14^ Situations such as a pandemic are complex, raising complex questions with often complex answers, highlighting the need to make space for a different paradigm. Epidemic preparedness and response as well as health systems strengthening initiatives are increasingly recognising epidemics as complex biosocial events.^17^ The complex systems theory has been recently proposed as one way of shifting the traditional positivist paradigm, reflecting more complex and dynamic relationships in the real world which needs to be addressed by a variety of methods,^14 18^ going beyond clinical trials. Others have also called for embracing the complementary insights achieved through diverse methods and diverse disciplines, sacrificing the need for a black and white picture of a problem for a more robust understanding of it.^19^

Most importantly, the debates around EBM have stopped being theoretical during the pandemic and challenged scientists in their day-to-day life as scientific advisors during the COVID-19 pandemic. In fact, some described the COVID-19 pandemic as the greatest challenge to EBM since the term has been coined,^20^ highlighting long-standing issues related to practising EBM, including rigour and independence of evidence, policy makers’ understanding of it, public understanding and trust in expert views, and managing ambivalence and contradictions in the societies.^4^

In the context of ‘the science’ becoming a focal point of this pandemic, scientific advisors have also found themselves in the public eye. While some gained a lot of recognition for their work, some have experienced personal attacks. This has been often magnified by politicisation of COVID-19, with media coverage playing a key role in how science and scientists have been perceived by the public,^21^ and the blurred boundaries between science, policy and politics,^4^ making it difficult for the public to make the distinction between scientific advice and government decision.

Preparedness for future health emergencies involves analysing the challenges and solutions employed during the current crisis; following the previous epidemics, including Ebola, SARS or Zika, numerous papers analysed country-specific or international responses to the situation (eg, refs 22–25).

While numerous papers have also been published analysing the challenges facing EBM and developing policies pre-COVID-19 pandemic (eg, refs 16 26 27) and during the COVID-19 pandemic (eg, refs 28–33), the views of key actors, that is, government scientific advisors related to COVID-19 pandemic, are still largely missing. This paper fills this gap by examining the views and experiences of scientists working on government advisory boards during the COVID-19 pandemic. The aim is to understand these experiences, to learn lessons for future pandemic preparedness and to understand how we can better support scientists working during future health emergencies.

## Methods

### Design

This is a qualitative study using semi-structured interviews. Qualitative designs are most suitable for understanding how people view, make sense and experience different phenomena,^34^ and this study aimed to explore how scientific advisors interpreted and experienced their new roles during the COVID-19 pandemic.

### Sampling and recruitment

We used purposive and snowball sampling to recruit scientists working on advisory boards during the COVID-19 pandemic. The inclusion criteria included (1) currently working at an academic or public health (research) institution and (2) experience of an official government advisory role during the COVID-19 pandemic in any of the following five European countries: Belgium, the Netherlands, UK, Sweden and Germany. These countries were chosen to provide variation in how boards operated. We aimed to recruit scientists from a range of disciplines mirroring the composition of boards across Europe.^4^ To recruit participants, three methods were used. First, we recruited participants through the European Union-funded Rapid European COVID-19 Emergency Response research (RECOVER) project, which was set up at the initial stage of the pandemic in February 2020 and included international scientists leading different biomedical work packages. The project was one of the first projects aimed to inform Europe’s response to the pandemic and any future emerging infectious disease outbreaks; this social science substudy was added to the portfolio of the wider project in December 2020. We recruited participants within this existing network but taking into account our inclusion criteria and purposefully selecting the proposed candidates. Second, we used snowballing sampling by asking RECOVER partners to reach out to potential participants from their own networks. Third, email invitations were sent to potential participants identified from government websites where member lists of COVID-19-related advisory boards were available. A letter of invitation, information about the study and consent form were emailed to all potential participants. All participants gave verbal consent to take part and for their interviews to be recorded. During the consent process, researchers discussed the sensitive nature of the study and highlighted that participants will have an opportunity to read the final draft of the findings and illustrative quotes (raw data) to ensure that they are not identifiable. All participants agreed to this process. Only information regarding the participant’s country is provided alongside the quotes to ensure anonymity. All transcripts have been de-identified and stored securely in line with the University of Oxford and University of Antwerp policies.

### Data collection

Two female experienced postdoctoral qualitative researchers (EC and MW) who had no working relations with the participants conducted video or telephone interviews. The interviewers followed a topic guide exploring the meaning of being a scientist working during the pandemic; new roles and responsibilities; experiences of working with other scientists within advisory boards, collaborating with governments; and informing the public (see online supplemental file 1). The topic guide was developed based on the existing literature and the research question, with the focus on four main areas: taking on new roles, informing policy, being in the public eye and working with other scientists. The interviews were conducted in English or Dutch, audio-recorded and transcribed verbatim. Field notes were made after each interview. Interviews continued until data saturation was reached.^35^

10.1136/bmjgh-2021-006928.supp1 Supplementary data

### Data analysis

A combination of inductive and deductive thematic analysis techniques was used.^36^ After conducting the first 10 interviews, the two interviewers read the transcripts to immerse themselves in the data. They then coded the data into 15 a priori categories based on the topic guide (deductive component). The deductive component was used to provide an initial framework for grouping data in relation to the research question and allowed researchers to quickly familiarise themselves with all data.^36^ To ensure that themes were grounded in the data, data within each category were then coded inductively line by line to create subcategories to create themes and subthemes. This thematic framework was discussed within the wider multidisciplinary team and then used to code the remaining interviews. In order to enhance the quality of the analysis, researcher triangulation and member checking were carried out.^37^ This involved discussion of the data and analysis at several stages among the wider multidisciplinary team, comprising psychologists, sociologists and a public health scientist. As part of the analysis process, we also sent a draft of the results to all participants for their feedback and to give them a chance to reflect on whether any important issues were missing. Given the sensitive nature of the study, we also made sure that all quotes were sufficiently anonymised and asked permission for using the quotes. NVivo V.12 was used to support the analysis process.

### Patient and public involvement

Given that patient and public involvement (PPI) was not central to the topic and the very rapid set-up of the study, PPI involvement was not possible.

## Results

Interviews with scientific advisors were undertaken between December 2020 and April 2021. The average length of the interviews was 43 min (range: 30–61 min).

In total, 84 scientific advisors were invited to participate from five countries. Twenty-one semi-structured interviews were carried out, resulting in a response rate of 25%. Three scientific advisors agreed to participate, but were not able to schedule an interview, and another three experts declined their participation, after having first agreed, due to concerns related to being identified. Many participants had ‘out-of-office’ email replies stating that they were working on scientific boards and would be unlikely to respond.

Table 1 gives an overview of the number of scientific advisors invited in each country. This varied depending on the availability of official member lists of advisory boards and the extensiveness of the network of the research team.

**Table 1:** Response rates across participating countries

	Belgium	The Netherlands	UK	Sweden	Germany	Total
Invited	9	10	53	6	6	84
Interviews conducted	5	6	7	2	1	21

Table 2 provides a brief description of all participants. Most participants were biomedical scientists (n=16), representing the fields of virology, immunology, microbiology, modelling, statistics, epidemiology, global health and public health. Five participants were social scientists representing the fields of psychology, sociology and behavioural science. The majority were solely employed at a university (n=17), while two participants worked at a public health (research) institution and two participants combined an academic position with a position in a governmental research institution. Majority of the participants were male (n=16).

**Table 2:** Overview of study participants

	Belgium n=5	The Netherlands n=6	UK n=7	Sweden n=2	Germany n=1	Total n=21
Male	4/5	5/6	5/7	1/2	1/2	16/21
Biomedical background	4/5	4/6	5/7	2/2	1/1	16/21
Seniority: full professors	5/5	6/6	6/7	1/2	1/1	19/21
Recruitment method	RECOVER network 2/5; snowballing 2/5; government website 1/5	RECOVER network 2/6; snowballing 4/6	Government website 7/7	Snowballing 2/2	RECOVER network 1/1	RECOVER network 5/21; snowballing 8/21; government website 8/21

RECOVER, Rapid European COVID-19 Emergency Response research project.

We identified five themes capturing participants’ views and experiences of working during the COVID-19 pandemic:

1. Complexities of working on scientific boards.
2. Learning to present evidence and recommendations.
3. Nature of the relationship between scientists and government.
4. Making sense of the boundaries of their role as a scientific advisor.
5. Being in the public eye.

These themes are discussed below with supporting quotes.

### Theme 1: complexities of working on scientific boards

Participants described numerous opportunities and challenges related to working on scientific boards.

Participants highlighted that scientific boards were composed of scientists representing different disciplines and that the key to success of this interdisciplinary collaboration was to focus on one’s expertise and respect each other’s specialist knowledge. They reported a great sense of satisfaction in working with their peers and described a sense of community among scientists who enjoyed these—in many cases—new collaborations. Equally, pre-existing collaborations seemed to facilitate good working relationships. Some noted that the pandemic facilitated more collaboration across universities than the prepandemic work, when relationships were more competitive, because participants were working towards the same goal of tackling the pandemic.

However, interdisciplinary collaboration within scientific boards did not always go smoothly. Integrating insights from various specialisms within a single board was also a challenge when individuals felt that their area of expertise should be a priority, and some participants felt that, at times, some of their colleagues overstepped their expertise, lacked openness towards other disciplines or even questioned the expertise of their peers, which resulted in frustrations.

[Talking about interdisciplinary collaboration] That was most frustrating because we were also sitting at the table with people who didn’t want to understand. You should at least be open to the vision of one another. (P19, Belgium)

Participants felt that, at the start of the pandemic, scientific boards tended to be dominated by colleagues from biomedical sciences, particularly virology. This meant that participants viewed the initial approach to tackle the pandemic as somewhat one-dimensional. Social scientists in particular reported difficulties in making their voice heard, by not being represented to the same extent as other disciplines. Participants felt that the composition of the board was very important in how ‘evidence was viewed’ and whether ‘it was accepted and taken forward’ within board decisions. They reported that the understanding of what constitutes ‘evidence’ seemed to differ among board members who were not always familiar with methods used by other disciplines. For example, some social scientists felt that they had to learn how to communicate their research and methodologies to their colleagues for their evidence to be seen as equally valid to that from biomedical disciplines.

What is striking is what they consider scientific evidence […], for example, we have different types of evidence for example, surveys or interviews and I really had to learn how to present this as legitimate evidence. […] I’d try to communicate in a way that people from that side would understand. […] think I even provided more background and detail than some of the medical things that are taken immediately as truth. (P16, UK)

However, over time, participants reported that it became apparent that a broad array of disciplines were needed to be able to inform the governments’ strategy as the pandemic had affected all aspects of society. With time, they also learnt from each other’s expertise, all having to broaden their scientific horizons, got to know each other better and built trusting relationships.

It keeps everyone on their toes, the motivational psychologists, the economists and so on, who gradually learn the more biomedical and bio-statistical side of the story. And vice versa, for example, you learn the important motivational elements in communication. (P14, Belgium)

### Theme 2: learning to present evidence and recommendations

Scientists described that working on advisory boards during the pandemic was different from how they worked prepandemic for a number of reasons.

First, they highlighted that different advisory boards had different ways of operating, which meant that the roles they took on varied as well. On some advisory boards, the scientists were tasked with generating and presenting evidence; on some with knowledge transfer based on their expertise in a particular field, while others involved giving recommendations or overseeing implementation of their advice.

So for example on X [name of scientific committee], the data and our opinions would be collected and we would have to say: ‘How certain are we of this opinion?’ So we would say: ‘Low certainty, medium certainty, high certainty.’ So for the Y [name of scientific committee], that was more to try to help ministers understand some of the concepts. And also, to say why we didn’t know […]. So there were different types of functions. (P10, UK)

Second, participants were often tasked with providing evidence and/or recommendations very rapidly. They highlighted that the ever-changing pandemic situation meant they were trying to gain a quick understanding of the emerging evidence, which was constantly changing. They worked very long hours to meet tight deadlines in order to provide evidence before it became outdated.

If you have results that are two weeks old, they are already a bit outdated and of limited use. And that is of course a completely different kind of speed […] during a pandemic you really need to find the balance between what are the most important things that you really want to get right (ie, quality), and what are those things you can deliver within those few weeks (ie, speed). (P21, the Netherlands)

You actually have to think very quickly, react very quickly and also develop your opinion very quickly, which of course contrasts with the slowness of science. (P15, Belgium)

Experts also faced difficulties when being tasked with making recommendations when evidence was limited or inconclusive. They highlighted that the prepandemic way of providing evidence involved feedback from colleagues, for example, peer review, and presenting it in a more nuanced way. In contrast, they were now tasked with providing an answer to specific questions and offering a more definitive answer, which challenged their previous ways of working. This also meant that, at times, public pressure affected recommendations by forcing scientists to make a recommendation despite limited or inconclusive evidence.

We have had endless discussions about those face masks on the street and our opinion was that you really have very little evidence for that. But there was a lot of social pressure, so advice was actually drawn up under social pressure. (P4, the Netherlands)

Because of this lack of evidence, participants felt that the recommendations were sometimes susceptible to questioning by other experts, opinion makers, government members or other stakeholders.

You work in such a crisis with a very high degree of uncertainty. And that is very difficult, isn’t it, because then you often get stories such as the fitness sector says “Yes, but where is the proof that we contributed to the increase in that second wave?” Well, you just can’t give that proof because you just can’t get that data out of the system. (P8, Belgium)

### Theme 3: nature of the relationship between scientists and government

Overall, scientific advisors felt that collaborations between advisory boards and government were somewhat working and that, in comparison with both how and the extent to which experts were consulted prepandemic, scientists had a more profound impact on policy decisions now.

Nevertheless, they also described tensions between advisory boards and policy makers and mentioned two main challenges. First, they highlighted that governments did not always take on board their evidence or did not take it on in a timely manner. Second, some participants felt that, at times, governments presented policy decisions as if they were solely based on scientific evidence, which they thought was misleading for the public. Some scientific advisors also felt that politicians were ‘using’ the advisors to justify political decisions in their communication to the public, especially related to unpopular restrictions.

At some point in March, our Prime Minister said “I do what the [name of advisory board] says”. I found that a very unpleasant comment, because it implies that you are directly responsible for the policy pursued and that is not the case. It is simply a political choice. And we only give advice. So they should take it as such and not hide behind that advice. (P4, the Netherlands)

Some highlighted that policy makers did not necessarily have a scientific background and hence lacked basic understanding of scientific methods and processes. However, similarly to their relationships with their peers on the boards, scientific advisors felt that, over time, collaborations had improved. They attributed this to policy makers gaining a better understanding of scientific methods and improved communication, despite their frequent lack of scientific background. Some experts also described that they learnt how to formulate their recommendations more clearly and in a more implementable and compelling way, for example, using the GRADE (Grading of Recommendations, Assessment, Development and Evaluations) criteria to present evidence, which improved the chances of it being used by policy makers. They also raised the importance of transparency around the content of recommendations made by advisory boards, which could be facilitated by publishing the minutes of the meetings, and emphasised that differences between advice delivered by advisory boards and policy decisions made by governments should be clear.

### Theme 4: making sense of the boundaries of their role as a scientific advisor

Scientists tried to make sense of what their role as a scientific advisor was and what the boundaries were. This seemed to be not only linked to how advisory boards operate (as described in theme 1), but also how they felt about their role. That meant that participants interpreted their role in different ways and differed in how they saw their relationship with governments.

As described in theme 1, scientific advisors were at times faced with situations when the evidence was not translated into policy. They expressed a variety of views in relation to how they felt about it and whether they had taken any action to ‘rectify’ this issue. Most scientific advisors expressed frustration and disappointment when they had faced such situations. Despite this, most participants viewed their role as ‘just’ providing evidence and recommendations to policy makers and felt that it was the responsibility of the government to make decisions.

And the politicians have to make the final decision because they see the whole picture. We must be very careful as experts not to become politicians and not to cross that line. (P2, Belgium)

I sometimes am surprised by the decisions that are being made by the politicians but, ultimately, it’s them who decide. […] We sometimes express a very strong view that, from the scientific point of view, our consensus is absolutely clear that we now have to introduce various lockdown measures and they just don’t happen. But I do understand that the government has a range of pressures upon them and that our scientific view is just one of them. […] They’re elected representatives and I’m not there to say that they’re wrong, but just to say what the point of view of scientists is. (P3, UK)

Consequently, this was reflected in how they coped with setting these boundaries. Many were reluctant to take an active stand when their advice was not followed. They described their reluctance to comment on political decisions in the media as they wished to maintain a good relationship with policy makers but also wanting to stand apart from political arguments, thus highlighting the attempts and difficulties of maintaining equilibrium. Finally, they were reluctant to question policy decisions publicly as they did not want to undermine the public trust in the implemented measures.

In addition, some scientific advisors stated that, at times, certain politicians had asked them to withhold from commenting on policy decisions. Not with standing these reasons to hold back from publicly commenting on policy decisions, participants also valued their independence and academic freedom and felt that they should always think about their academic position and what that actually entailed. Consequently, in some cases, experts actively expressed their concerns in the media and tried to highlight the scientific evidence on the contested issue in order to achieve some kind of equilibrium between protecting and using their academic freedom, while maintaining a constructive attitude towards the government, which was not always easy. They highlighted that the role of academic advisors brought these issues to the forefront.

That is an equilibrium that you have to maintain: on the one hand, remain critical and have the feeling that you are able to express your thoughts as a scientist, but at the same time, you cannot be diametrically opposed to politicians. (P8, Belgium)

We have always said: we must be able to continue to communicate from a position of academic freedom. That is important. It has also made that your words weigh more heavily when you are a member of a board, and that is something you have to learn to take into account. (P14, Belgium)

Scientific advisors felt more inclined to influence policy makers through the media in cases when they felt that the channels of communication between scientists and governments were not working well and their recommendations concerning some crucial topics were not turned into policy. In these situations, they felt that media outlets could offer a solution to make sure that the public received the information they deemed necessary and to influence policy makers indirectly, by making their voice heard. Many highlighted that, in these occasions, they were speaking in a personal capacity rather than on behalf of a scientific board.

I wrote an article […]. Again, to bring this to public awareness. Again, to try and influence policy and get schools to reopen, and again just to bring it to public awareness, my concern about the ongoing psychological impact on mental health and well-being. So yeah, again it was just trying to get people to think, to engage, and again, to bring [my field] to the public awareness. (P18, UK)

### Theme 5: finding oneself in the public eye

Participants reflected on their motivations for joining advisory boards. They felt a sense of responsibility to use their expertise to help tackle the COVID-19 pandemic, which felt rewarding. This was to some extent reflected in how they viewed their role of communicating with the public. They described taking part in media interviews with the aim to ensure that the public were informed of new developments and scientific insights. Especially at the beginning of the pandemic, participants considered educating and informing the public about the COVID-19 virus and the pandemic as a key part of their role as a scientist.

In the first half, there was a great need for information. People didn’t really understand what was happening. And people wanted explanations. […] I thought it was great that I could contribute, […] that I could give that explanation to people. (P20, the Netherlands)

Multiple experts highlighted that, over time, the content of their media contributions shifted from explaining scientific insights towards explaining the measures that were taken to tackle the pandemic. Participants felt that it was important to have a meaningful role in public engagement to ensure that the public had access to correct evidence-based information to tackle ‘fake news’ and disinformation.

I believe we have to go on the media to put the point of the scientists across because unfortunately, there are things on social media that are just fake news and disinformation. […] I think it’s the duty of scientists to put the truth across as far as we know it. (P10, UK)

However, as a result, many experts found themselves in the public eye and consequently became very well-known figures. Developing a relationship with the media was one of the main challenges, with participants describing both successful and less successful examples. Some journalists were keen to hear from scientists and worked hard to make sure that science was described accurately by offering a platform and working closely together with scientists.

Participants also experienced less successful examples of media relations where media organisations played different scientists off against each other or against policy makers (sometimes without prior warning), used quotes taken out of context, and called them unexpectedly and pressured them into providing a more black and white picture of the situation than they were prepared to present, thus highlighting the complex relationship between multiple actors in this pandemic: the public, the media, the scientists and the government.

Many highlighted that engaging with the media took a lot of time and that media attention was excessive. Some scientists also highlighted that presenting scientific evidence is a skill and that the public should be made aware that science evolves. While the current scientific evidence points in one direction, future research can challenge previous assumptions and scientists need to adapt their thinking and communicate these developments to the public.

You really need to understand and live with the knowledge that this is constantly changing. You can never be quite sure that what science delivers to you one month, or the next month. And you need to be transparent about that when you communicate about it. (P6, Sweden)

Some participants highlighted that the press created a particular narrative around government decisions linking individual scientists to certain political decisions, as did some politicians (as described in the theme 3). Because of this, certain scientists were perceived by the public as responsible for government decisions.

The perception of responsibility is entirely wrong, because political decisions always take science as one of their inputs, while sometimes the public perceives political decisions to be entirely destined by scientific information, which is just not the case. […] Because of the picture that the media creates, it’s really a narrative in the media. (P7, Germany)

Consequently, many participants described positive responses from the public, which included getting ‘thank-you’ messages, while others also described receiving numerous negative messages, including death threats.

I have gotten a lot of death threats. So I get them emailed to me but I even get people calling work, you know agitated trying to find me. It’s quite scary actually so I had to make police report. (P16, UK)

## Discussion

Our study showed that scientific advisors found the experience of working during the pandemic rewarding. However, they also faced numerous challenges, including learning to work in an interdisciplinary way, ensuring that evidence is understood and taken on board by governments, and dealing with media and communication with the public. Scientists found themselves taking on new roles, the attributes of which were not clearly defined, and thus were interpreted in various ways. Because of these new roles, scientists received much media attention and were often perceived and treated as a public figure. This study highlights several lessons which can facilitate preparedness for future health emergencies.

### Strengths and weaknesses of the study

To our knowledge, this is the first European qualitative study exploring the experiences of scientists working on government advisory boards during the COVID-19 pandemic, thus giving a voice to key actors in this pandemic. The heterogeneity of our sample, which consisted of both medical and social scientists in five European countries, has enabled us to examine a variety of perspectives. We also note some limitations. First, the limited number of participants from some countries prevented cross-country comparisons. It was encouraging though that scientists’ views within and between countries have largely been consistent, highlighting that the key tensions and opportunities of working as a scientist were shared between contexts. Second, the study focused on Europe and thus the experiences of scientists in this study may not be transferable to other regions, both in high-income and low-income countries. Third, the current study only describes the views and experiences of scientists advising policy makers at a national level. In addition, giving a voice to policy makers could also lead to valuable lessons on how to improve the collaboration between scientists and policy makers. Fourth, interviews with scientists were conducted during the second and/or third waves in their respective countries. Interviewing scientists during the first wave might have shed a different light on their experiences; however, by asking them to reflect on what has changed, we were able to capture some of their views of working during the initial stages of the pandemic retrospectively. Fifth, majority of the participants were male and from biomedical fields. While our sample reflects under-representation of women^38 39^ and non-biomedical disciplines in scientific advisory panels in general,^40^ future studies could explore these issues, including the power dynamics going beyond the disciplines described in this study. Sixth, the sample may to some extent reflect the wider RECOVER project’s own network and the fact that in most countries the lists of scientists advising the government are not easily available. Finally, it is important to be reflexive on the role of the research team in relation to the topic of the study; as the researchers conducting the interviews were scientists themselves, who on a smaller scale also responded to the COVID-19 pandemic, they have regularly reflected on their own perceptions and experiences of working during the pandemic to ensure the accurate representation of participants’ voices. It is important to highlight that the interviewers have not had pre-existing working relationships with the participants. They have also made reflective notes after each interview, regularly discussing the analysis within the team and continuously getting back to original data to check one’s interpretations and understanding.

### Comparison with other studies

Our study highlighted a number of challenges facing scientists and the complexities of power between scientists, policy makers, media and public. First, the scientists in our study highlighted issues related to the cross-disciplinary and interdisciplinary collaboration within scientific advisory boards, which was not always successful. They felt that especially at the start of the pandemic, there were difficulties with integrating the insights from the various specialisms. The issue of difficulties of working with other disciplines is not new to the COVID-19 pandemic (eg, refs 17 41) but seemed to have been brought to the forefront during the current health emergency. Others also raised the importance and difficulties of cross-disciplinary collaboration in the context of practising healthcare during the pandemic^42^ and policy making.^43^ Other authors also elaborated on this issue by, for example, highlighting that what counts as a ‘fact’ differs across disciplines, including what we understand as evidence and what counts as reliable data and methods of data collection.^44^ We found that scientists felt that establishing new relationships takes time and the limited pre-existing interdisciplinary ties posed a challenge for scientists working during this pandemic within scientific boards in which a wide spectrum of specialisms were represented.

Second, our study highlighted numerous challenges facing scientists when producing and presenting emerging and changing evidence to policy makers. This included the pace of work, limitations of available evidence yet being put under pressure to provide policy recommendations, and evidence or methods being questioned by policy makers. Altogether, it also highlighted that the traditional way of producing, assessing and implementing evidence was not always compatible with the challenges posed by the pandemic, namely the urgency of addressing the situation. These challenges have been somewhat acknowledged previously^28 44^; for example, Cairney and Wellstead^28^ highlighted that one of the key ingredients of successful policy making is for policy makers to have trust in experts and their recommendations. Indeed, the pandemic challenged previous ways of working not only for scientists but also policy makers, highlighting that adoption of innovations,^45^ that is, working with others from fields, disciplines or methods not familiar to them, is challenging. Our study highlighted what these challenges meant for the day-to-day life of scientists during this pandemic.

Third, our study also highlighted that the role of scientific advisors was somewhat unclear. This was linked to both the way boards operated but also how, particularly at the start of the pandemic, scientists were portrayed by both media and policy makers as responsible for policy decisions. In line with previous studies describing theoretical analyses of policy-making process,^3 9^ participants described that, at times, their recommendations were positioned by governments as the sole basis for decisions and felt that policy makers used experts to justify their decisions. For policy makers, aligning with science is considered an effective risk communication strategy.^46^ It becomes problematic, however, when decision-making is clearly not in line with recommendations made by the scientific advisory boards, which has occurred on various occasions, as described by the participants in this study and others.^9^ This study is the first to explore how, within this confusing context, scientists made sense of their role and in fact had varied interpretations of it. While the majority felt they should withhold from publicly commenting on policy decisions, some felt that they should speak up when key recommendations were not taken on board. Moreover, at times, our participants highlighted that policy makers have pressured them to not discuss certain issues in the media, while the media has often urged them to comment on policy decisions.

Finally, our study raised the issue of the labour required from the scientists to engage with the media and the public, which also made scientists vulnerable to attacks. The importance of scientists engaging with the public is well recognised,^47 48^ and the study participants valued and saw it as an important aspect of their role as scientific advisors; however, this study provided a detailed picture of scientists’ experiences of working with the media during this pandemic.

### Lessons learnt and policy implications

Our study identified a number of lessons aimed at policy makers, public, higher education and scientists themselves which can facilitate preparedness for future health emergencies. These are summarised in table 3.

**Table 3:** Summary of recommendations in relation to key issues

Key issues	Target audience	Recommendations
Need for interdisciplinary collaboration	Policy makers	Provide and strengthen entry points for all relevant disciplines in responding to the pandemic or health emergency, such as behavioural, social and political sciences, engineering, and economics, bringing added value to clinically and biomedically oriented work.
	Scientists and higher education system	Provide training and promote scientists’ development of skills facilitating interdisciplinary working early on in higher education system and as part of professional development.
Importance of clear role boundaries for scientists on government advisory boards	Policy makers	Provide clear definitions of advisory and decision-making roles, alongside a transparent roadmap of responsibilities, tasks and procedures, in order to facilitate and enhance effective and trustworthy science advisory process.
Dealing with emerging and changing ‘evidence’ while providing recommendations	Scientists and policy makers	Promote clear process of assessing the strength of available evidence. Identify strategies to tackle misinformation and disinformation, including during the pandemic as well as long term, through investment in adult and public education on scientific methods and limitations of science.
	Scientists	Communicate transparently scientific uncertainties and be explicit in order to maintain trustworthiness and facilitate public trust in science.
Clear communication of science to the public	Scientists	Communicate current scientific understanding in an approachable, clear and transparent way, separate from political communication.

First, our findings highlight that further development of scientists’ skills is needed to promote interdisciplinary collaboration. While governments focused on understanding the nature and epidemiology of the virus,^49^ other disciplines could have made important contributions related to questions around social practices related to transmission of the virus, disease perception or help-seeking behaviour, as has been shown in other outbreaks.^50^ Looking for entry points where disciplines such as behavioural, social and political sciences, engineering, and economics can bring added value to some of the more clinical or biomedically oriented work can improve advisory boards’ preparedness to perform their expert roles during future crises.^17^ As measures to tackle a pandemic touch every aspect of society, it is key to involve experts of a wide range of disciplines who have the skills to work in an interdisciplinary fashion.^4 51^ The importance of interdisciplinarity also needs to be promoted in higher education, with the explicit aim of embracing interdisciplinarity, rather than promoting discrete professions,^52^ thus preparing new generations of scientists not working in silos. As researchers experience numerous systemic barriers to work in a cross-disciplinary way, such as career prospects,^53^ these need to be tackled as well. Some suggested that one way of facilitating interdisciplinarity might be to focus on developing understanding of methods used across disciplines, as they are often what unify or separate scientists.^54^ Given the complexity of the problems posed by situations such as health emergencies, the methods themselves also need to continuously develop to fit the context and resources.^54^ Second, this study has highlighted a need to better clarify the role of scientific advisors towards policy makers, media and the broad public. Clear definitions of advisory and decision-making roles should be provided, as well as a clear roadmap of responsibilities, tasks and procedures, to protect the boundaries of the expert role and to facilitate an effective and trustworthy science advisory process.^4 6 50^ Third, our study showed that scientists at the heart of the fight against COVID-19 invested time in regular and social media contact to disseminate accurate and timely information. In doing so they hoped to help tackle misinformation and disinformation, which has already been named an ‘infodemic’.^52 55–57^ It has indeed been highlighted that communication of science to the public is a responsibility of scientists^58 59^ and higher education should facilitate researchers’ development of skills needed to interact with the public,^59 60^ while also making sure that scientists see the value of interacting with the public.^61 62^ However, facilitating public understanding of science is also an important task for public health institutions and governments,^63 64^ and more research is needed to identify effective strategies to tackle misinformation and disinformation.^63^ Fourth, there is also a need to support the public in developing skills through novel educational programmes oriented to teach citizens the strengths and limitations of scientific practices. Fifth, our study highlighted the need for scientists for scientific uncertainties to be explicitly assessed and communicated transparently to the public in order to maintain science trustworthiness and facilitate public trust in science.^4 51 65^ The competence to communicate science in an approachable, clear and transparent way^56^ as factual, transparent communication, which is separated from political communication, is key during a crisis.^56^ Finally, the pandemic also highlighted potential shortcomings in higher education system to prepare the academic community to work in health emergencies and take on new roles and responsibilities, including dealing with media, speaking to the public and giving policy recommendations. It is important that these are developed and are an important part of the curriculum in higher education and as part of professional development, with this pandemic highlighting the benefits of more transferable skills as well.

## Conclusions

Since January 2020, scientists have played a key role in tackling the COVID-19 pandemic. While many countries have started to evaluate their COVID-19 response, it is crucial that we take on board key lessons shared by scientific advisors, which call for building interdisciplinary collaboration within advisory boards; ensuring transparency in both how boards operate and define and protect the boundaries of the scientist–government relationship; and supporting scientists in their role to inform the public. While there may not be easy solutions to all these issues, countries may learn from each other’s experiences in how to increase transparency in their decision-making processes and protect the trustworthiness of scientific advisors.

## Data Availability

No data are available.
