# Supplementary material for: Following the science? Views from scientists on government advisory boards during the COVID-19 pandemic: a qualitative interview study in five European countries
Source: BMJ Glob Health. 2021 Sep 27;6(9):e006928. doi: 10.1136/bmjgh-2021-006928 (PMC8478576; doi:10.1136/bmjgh-2021-006928)
Supplement: Supplementary data [file bmjgh-2021-006928supp001.pdf]

Exploring the views and experiences of scientists working in the public eye on COVID-19 research.

Topics to be explored

Below is a list of topics to be discussed. The topic guide will remain flexible with respect to what is of importance to participants.

1. **What does it mean for you to be a scientist working in the public eye on COVID-19?**

2. **Roles & responsibilities**
   What additional roles or responsibilities have you taken on as a result of the pandemic and how have you found these?
   How did the roles come about? What was your motivation for taking this role? How have you found the role in terms of what you initially expected?

3. **Experience of sharing evidence and informing policy**
   i. *Informing and collaborating with policy makers*
   How have you been involved in presenting evidence to inform policy and how have you found this?
   - How have you found collaborating with government and policy organisations? How have these collaborations evolved over time?
   -

   ii. *Informing the public:*
   How have you been involved in presenting scientific evidence to the public and how have you found this?
   - How has your role in informing the public about COVID-19 evolved over time?
   - How do you feel about how the press covers your scientific contributions?
   - How do you feel about sharing your scientific evidence on social media?
   - How do you feel about the way the government presents (your) scientific evidence to the public?
   - What kind of reactions have you received from the public? Have you received disturbing reactions or threats? How did you deal with this?
   -

   iii. *Collaborating with other scientists*
   Has COVID research led you to collaborate with new scientists and if so how have you found this?
   - What has been going well? what has been more difficult in these collaborations?
   - How have these collaborations evolved over time?
   - Have you experienced tensions or conflicts with other researchers?

4. **Overall experience**
   - **How has your involvement in COVID-19 research and advising policy/government affected your professional life?**
     How do your COVID-19 roles and responsibilities impact your existing roles?
   - **How has your involvement in COVID-19 research and advising policy/government affected your personal life?** How have you dealt with that?

5. **Lessons learned**
   What have you learned from your experience of informing COVID policy? What do you think are the most important lessons from this experience?
   - If a new pandemic breaks out in the future, what should be done differently in terms of bringing scientists and policy makers together?

Concluding questions:

- Do you have any additional remarks?
- Is there something that you think we didn’t cover that is relevant to this issue / topic?
- Is there someone else you think we should talk to?
